# Cycling hypoxia affects cell invasion and proliferation through direct regulation of claudin1 / claudin7 expression, and indirect regulation of P18 through claudin7

**DOI:** 10.18632/oncotarget.14397

**Published:** 2016-12-31

**Authors:** Hong Liu, Feifei Jiang, Xinshan Jia, Jing Lan, Hao Guo, Erran Li, Aihui Yan, Yan Wang

**Affiliations:** ^1^ Department of Otolaryngology, The First Affiliated Hospital of China Medical University, Shenyang, Liaoning 110001, China; ^2^ Department of Pathology, China Medical University, Shenyang, Liaoning 110001, China; ^3^ Department of Dermatology, China Medical University, Shenyang, Liaoning 110001, China; ^4^ Institute of Respiratory Disease, The First Affiliated Hospital of China Medical University, Shenyang, Liaoning 110001, China

**Keywords:** claudin1, claudin7, HIF1α, cycling hypoxia, nasopharyngeal carcinoma

## Abstract

Claudins (*CLDNs*), the major integral membrane proteins at tight junction, play critical roles in apical cell-to-cell adhesion, maintenance of epithelial polarity, and formation of impermeable barriers between epithelial cells.

We investigated in this study the expression of *CLDNs-* Claudin1 (*CLDN1*) and Claudin7 (*CLDN7*), and their relation to tumor progression in nasopharyngeal cancer (NPC). *CLDN7*, rather than *CLDN1*, showed higher expression in both undifferentiated tumor tissue and the poorly differentiated CNE2 cells, compared with differentiated tissue and the highly differentiated CNE1 cells. Furthermore, knockdown of *CLDN7* dramatically inhibited the metastasis and invasion of CNE2 cells suggesting that *CLDN7* could act as a biomarker for NPC metastasis.

Cycling hypoxia could induce significant changes in *CLDN1* and *CLDN7* expression in NPC cells. Genetics analysis demonstrated that *CLDN1/CLDN7* were not only regulated directly by HIF1a but also affected each other through a feedback mechanism. *CLDN7* acted as a bridge to promote HIF1a-induced P18 expression and cell differentiation. Taken together, our results provide evidence that adjusting the oxygenation time and cycles in NPC might be an effective method to prevent / delay the metastasis of poorly differentiated NPC cells.

## INTRODUCTION

Claudins (*CLDNs*) play an essential role in the function of the tight junction (TJ), and the maintenance of the polarity of epithelial cells. So far, 24 *CLDNs* subtypes have been identified [1–3]. Previous studies have identified the expression of *CLDNs* in several cancer types, and demonstrated that even the same *CLDN* subtype might have different functions in tumorigenesis and metastasis in different cancers. Several papers define the ultrastructural anatomy of the tight junction and suggest that a single tight junction with differences in protein composition and structure in different subdomains [4–5]. Some of the *CLDNs* also can form strands in other non-epithelial cell or be found outside of TJ [6–8], where their functions are still disputed.

*CLDN1* has been predicted to act as a tumor suppressor gene in carcinomas of breast, prostate, colon, and liver [9–15]. However, the high expression of *CLDN1* can mediate TNFα-induced gene expression, promote cell invasion and inhibit apoptosis in human gastric adenocarcinoma MKN28 cells, MCF7 breast cancer cells and A549 lung cancer cells [16–18]. In nasopharyngeal carcinoma (NPC) cells, up-regulated *CLDN1* expression confers resistance to cell death [19].

A lack of *CLDN7* is a strong indicator of regional recurrence in oral and oropharyngeal squamous cell carcinoma [20]. However, in ovarian carcinoma, CLDN7 is significantly up-regulated and may be functionally involved in ovarian carcinoma metastasis [21]. *CLDN7* over expression in the human gastric adenocarcinoma cell line AGS can increases its invasiveness, migration, and proliferation. *CLDN7* can form a complex with EpCAM, CD44 variant isoforms, and tetraspanins to promote colorectal cancer progression [22, 23]. In NPC, *CLDN7* overexpression is associated with metastasis and a low survival rate [24, 25]. Several studies further reported that *CLDN7* had polymerization tendency and can be found outside of TJ [26], and that the role of *CLDN7* in tumor was associated with their polymerization and localization status inside the cells [26, 27].

Clinical studies have shown that 100% of primary NPCs and 58% of cervical nodal metastases of NPCs contain hypoxic regions [28]. HIF1α protein is over expressed in NPC tissues compared with normal nasopharyngeal tissues, and plays a major role in tumor development, including growth rate, invasiveness, angiogenesis, and metastasis [29]. However, the effect of hypoxia on the expression of *CLDNs* in NPCs remains unknown.

The present study aimed to evaluate the expression of *CLDN1* and *CLDN7* under different cell differentiation status, and their relationship to tumor progression in NPCs. The impact of hypoxia on *CLDN1* and *CLDN7* expression was also evaluated in a hypoxicmodel.

## RESULTS

### The *CLDN1/CLDN7* expression are correlated to the differentiation status of the nasopharyngeal cancer

**Figure 1:**
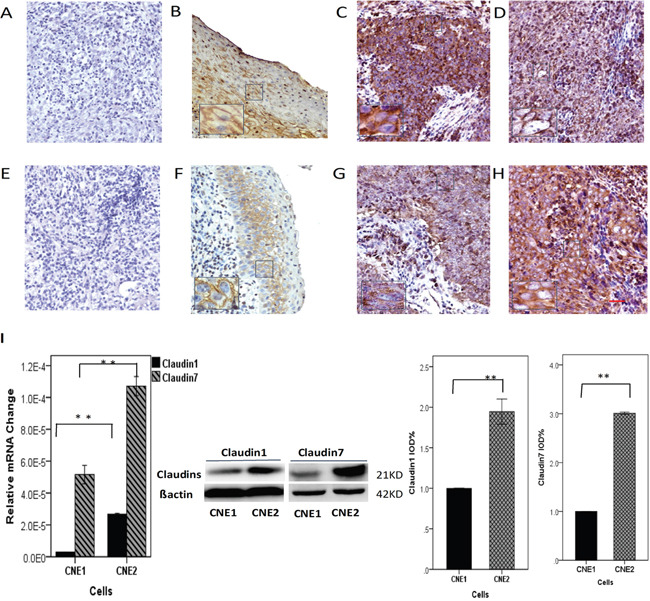
Brown staining demonstrates the expression and location of CLDN1 **A-D**. / **CLDN7 (E-H)** in nasopharyngeal carcinoma (NPC), and only membranous and/or cytoplasmic staining was classified as positive. **A, E**. Negative control of *CLDN1/CLDN7*. **B, F**. *CLDN1/CLDN7* were highly expressed in the stratified squamous nasopharyngeal epithelium. **C, G**. *CLDN1/CLDN7* were highly expressed in well-differentiated NPC tissues. **D, H**. *CLDN1/CLDN7* were highly expressed in poorly differentiated NPC tissue. *CLDN7* showed a much increased expression rate in the poorly differentiated NPC tissues **H**. compared to the well-differentiated NPCs **G. I**. Comparing the expression of *CLDN1/CLDN7* in CNE1 and CNE2 cells based by RT-PCR and Western blotting: both were highly expressed in CNE2. Scale bar = 100 μm. **: P<0.05.

The samples were divided into two groups: low *CLDN1/CLDN7* expression (score of 0 to 2) or high *CLDN1/CLDN7* expression (score of 3 to 9) samples. As shown in Figure 1, *CLDN1* expression rate was high at 65.6% (25/38, Figure 1C) and 68% (17/25, Figure 1D) in differentiated and undifferentiated NPC specimens, respectively. *CLDN7* expression rate was shown at 42.5% (17/40, Figure 1G) and 61.5% (16/26, Figure 1H) in the differentiated vs. undifferentiated NPC specimens, respectively. *CLDN7* expression was negatively correlated with the differentiation status of the nasopharyngeal squamous cell carcinoma, with a higher expression in undifferentiated NPC samples (Figure 1H).

### Correlation between *CLDN1/CLDN7* expression and nasopharyngeal cancer cell differentiation

We next used CNE1/CNE2 cells to further confirm the result above. CNE1/CNE2 cells represent well-differentiated and poorly differentiated NPC cells, respectively. We evaluated the correlation between *CLDN1/CLDN7* expression and the differentiation status of the cells. The real-time PCR (for primer sequences, see Table 1) and Western blot results showed that there were significantly higher expression of *CLDN1/CLDN7* in CNE2 than in CNE1 (Figure 1I).

**Table 1:** Primers used for PCR

Gene	Sense	Antisense
*CLDN1*	5′-CCCTATGACCCCAGTCAATG-3′	5′-ACCTCCCAGAAGGCAGAGA-3′
*CLDN7*	5′-AGAGCACTTTGGACAGAACCC -3′	5′- CTCCGGACTGGATTTCCCTC-3′
P18	5′-GCCGAGCCTCCTTAAAACTC-3′	5′- GAGGGTGCCGGTTTCTTCT -3′
HIF1α	5′- ACCTATGACCTGCTTGGTGC -3′	5′- GGCTGTGTCGACTGAGGAAA -3′
β-actin	5′-TGGCACCCAGCACAATGAA-3′	5′-CTAAGTCATAGTCCGCCTAGAAGCA-3

Combined with the immunohistochemical staining data above, *CLDN7* demonstrated that the expression in poorly differentiated carcinoma was significantly higher, suggesting a close association with the differentiation of NPC tissue and cells. Because the poor differentiation of cancer is generally considered to be related to high metastasis and low survival rate, therefore we took the next step to investigate the correlation of CLDN7 expression with the invasion of NPC.

### *CLDN7* promotes NPC invasion and migration

**Figure 2:**
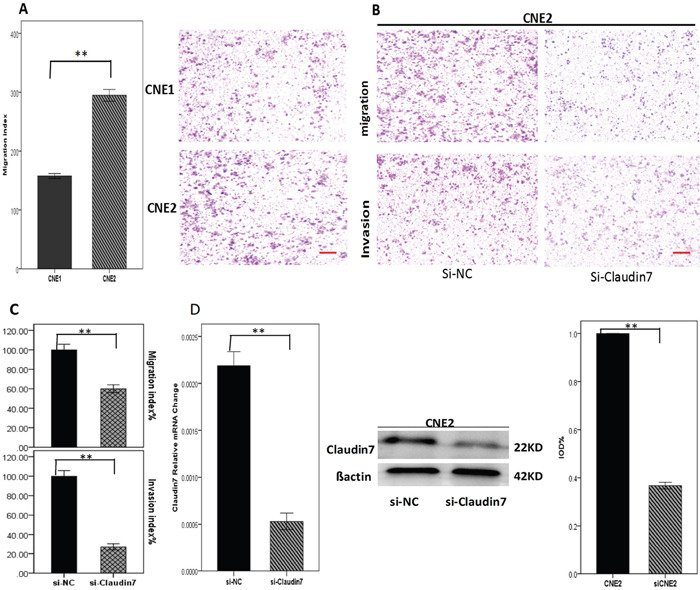
**A**. Migration index analysis of CNE1 and CNE2 using transwell model showed that CNE2 cells with high *CLDN7* expression had a significantly increased migration ability than CNE1 cells. **D**. RT-PCR and Western blotting showed the results of knocking down *CLDN7* in CNE2; the Si-RNA silencing efficiency was approximately 60-70%. **B, C**. Migration and invasion results of Si-NC and si-Claudin7 in CNE2 demonstrated that knocking down the *CLDN7* expression inhibited the migration and invasion ability of CNE2 cells. Si-NC is the negative control of silencing the expression of *CLDN7* in CNE2. All statistical analysis were compared to the control. Scale bar = 100μm. **: P<0.01.

CNE2 cells with high *CLDN7* expression, indeed demonstrated a greater migration capacity compared to CNE1 cells (Figure 2A), which supports the hypothesis that cells with poor differentiation status have high tendency of migration and invasion. Using small interfering RNA technology (for the si-RNA silencing gene sequences, see Table 2), we knocked down *CLDN7* in CNE2 to investigate the correlation between *CLDN7* and the cell invasion capacity. The result showed that the cell invasion and migration index was significantly decreased after si-RNA transfection for 24-48 h (Figure 2B & 2C), suggesting that *CLDN7* could enhance the metastasis of NPC.

**Table 2:** siRNA sequences

Gene	Sense	Anti-sense
si-HIF1α	5′-GGCCGCUCAAUUUAUGAAUTT-3′	5′-AUUCAUAAAUUGAGCGGCCTT-3′
si-*CLDN1*	5′-CCAUGGGUGGAGGCAUAAUTT-3′	5′-AUUAUGCCUCCACCCAUGGTT-3′
si-*CLDN7*	5′-GGCAUAAUUUUCAUCGUGGTT-3′	5′-CCACGAUGAAAAUUAUGCCTT-3′
si-con	5′-UUCUCCGAACGUGUCACGUTT-3′	5′-ACGUGACACGUUCGGAGAATT-3′

### The different responses of CNE1/CNE2 cells to hypoxia is associated with their differentiation status

**Figure 3:**
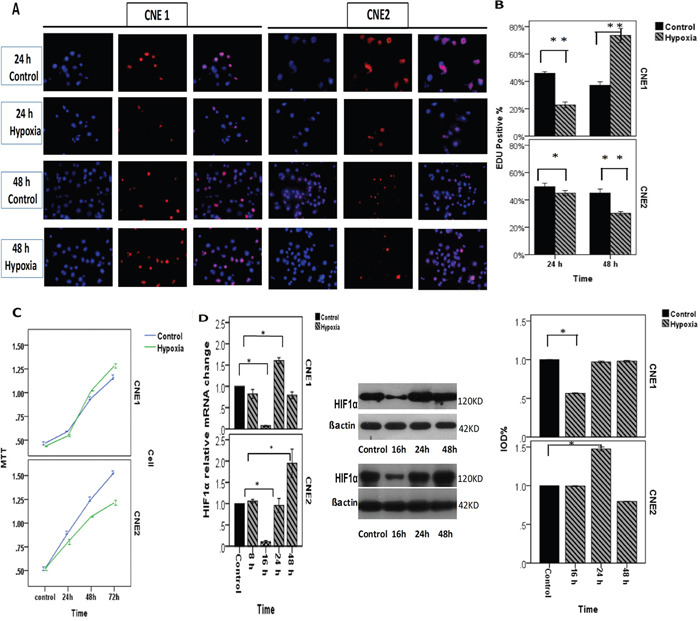
NPCs incubated in a hypoxic atmosphere of 0.1% O2, 5% CO2, and 94% N2 at 37°C for different amounts of time **A, B**. EdU of CNE1 shows that hypoxic conditions inhibited CNE1 proliferation before 24 h, whereas it may be promoted after 24 h. EdU of CNE2 shows that hypoxic conditions inhibited CNE2 proliferation. **C**. MTT of CNE1 shows the cell viability, and hypoxic conditions inhibited it at 24 h, whereas it may be promoted after 24 h. The MTT assay of CNE2 showed that hypoxic conditions can significantly inhibit cell viability and decrease the migration and invasion ability of CNE1 cells. **D**. RT-PCR showed the HIF1α relative mRNA change in CNE1, which experienced a down-regulation at 16 h and then increased and reached a peak at 24 h. RT-PCR showed the HIF1α relative mRNA change in CNE2, which experienced a decline at 16 h and then increased gradually and reached the peak at 48 h. The HIF1α protein change in CNE1, which decreased at 16 h significantly. Although the mRNA was increased at 24 h, HIF1α protein was not significantly changedcomparedwith the control. HIF1α protein change in CNE2, which decreased at 16 h then increased and reached the peak at 48 h gradually, which was higher than control.

**Figure 4:**
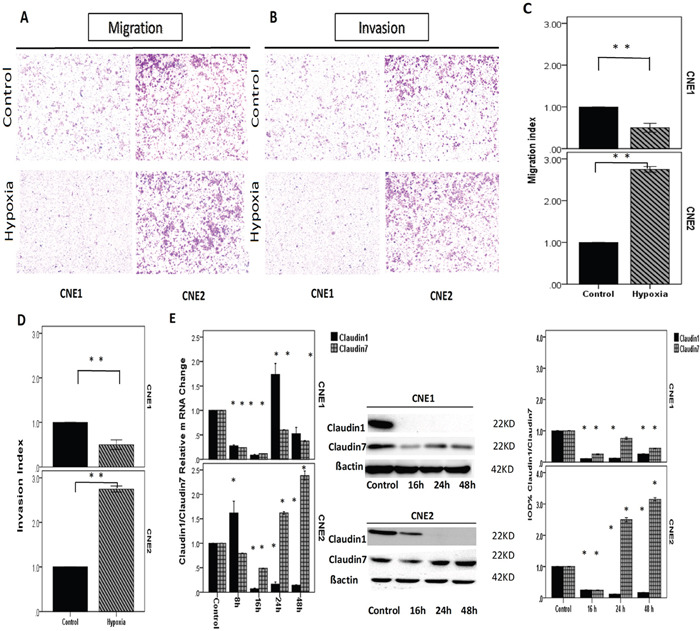
NPCs incubated in a hypoxic atmosphere of 0.1% O2, 5% CO2, and 94% N2 at 37°C for different amounts of time **A, B**. Images of the migration and invasion experiments of the NPCs conducted using transwell model. Compared with the control, the number of cells was significantly reduced in the hypoxic conditions in CNE1 cells and significantly increased in CNE2 cells. **C, D**. Bar graphs showing the transwell experiment results of CNE1 and CNE2. **E**. RT-PCR showed the *CLDN1/CLDN7* relative mRNA change in CNE1, and both experienced decreases before 16 h and then increased. The mRNA of *CLDN1* increased sharply and reached the peak at 24 h, which was two-fold higher than the control, but it declined significantly thereafter. *CLDN7* expression gradually increased, but it did not reach the control level by 48 h. The *CLDN1/CLDN7* relative mRNA change in CNE2 cells. *CLDN1* increased and reached the highest level at 8 h, after which it declined sharply at 16 h, 24 h and 48 h. *CLDN7* decreased at 8 h and 16 h then increased to higher expression than the control at 24 h and reached the peak at 48 h. The proteins of *CLDN1* and *CLDN7* in CNE1 cells both decreased significantly compared with the control. Although the mRNA of *CLDN1* was increased at 24 h, the protein did not increase after that point. The proteins of *CLDN1 and CLDN7* in CNE2 both decreased at 16 h then increased at 24 h and reached the peak at 48 h. All positive. *: P<0.05, all data were compared to the control.

NPC cells were incubated in a hypoxic atmosphere of 0.1% O_2_, 5% CO_2_, and 94% N_2_ at 37°C for 8, 16, 24 or 48 h. HIF1α expression was investigated using real-time PCR and Western blotting (Figure 3D). Cell proliferation and viability were then assessed using EdU and MTT assay (Figure 3A, 3B, 3C). The transwell analysis was performed to analyze NPC migration and invasion (Figure 4A, 4B). As a control, the cells were incubated at normoxic conditions of 20% O_2_, 5% CO_2_, and 75% N_2_. The results showed that the different reactions of CNE1/CNE2 cells to hypoxia are associated with their differentiation status. CNE2, as a relatively poorly differentiated NPC cell line, was more sensitive to hypoxia than CNE1. The expression levels of HIF1α mRNA and protein in CNE2 were up-regulated under hypoxic conditions at 24 h and 48 h, and CNE1 cells exhibited a similar trend in mRNA levels. However, HIF1α protein levels were slightly reduced at 24 h and 48 h in CNE1 cells. Hypoxic conditions inhibited cell proliferation, and enhanced the migration and invasion ability of CNE2 cells; but accelerated cell proliferation and decreased the migration and invasion ability of CNE1 cells (Figure 3).

### Hypoxia altered *CLDN1/CLDN7* expression in NPCs

HIF1α is considered an independent prognostic indicator [28, 29] and is closely related to the invasion of NPC. Clinical studies have shown that 100% of primary NPC tumors and 58% of cervical nodal NPC metastasis contain hypoxic regions [28]. Under a hypoxic environment, the changes of *CLDN* proteins, which are important components of the cell membrane, could also potentially affect the NPC development including metastasis.

To investigate the impact of hypoxia in the expression of *CLDN1/CLDN7* in NPCs, we examined the expression variations of their mRNA and protein levels after cells were exposed to hypoxic conditions for different duration of time. Although the protein expression of *CLDN1* significantly decreased in both cells, there was a slight up-regulation in the mRNA levels at 24 h in CNE1 cells and 8 h in CNE2 cells. Compared with CNE2 cells, the *CLDN1/CLDN7* proteins were down-regulated more sharply in CNE1, and with *CLDN1* barely detectable from 16 h post hypoxia exposure (Figure 4E).

Hypoxic condition triggered a decline in the *CLDN7* protein production in CNE1 and increased the expression in CNE2. Real-time PCR reflected the relative mRNA change more precisely, and the changes of *CLDN1* and *CLDN7* were opposite in CNE2 cells (Figure 4E). *CLDN7* and HIF1α exhibited the same trend in both cells, which might be associated with their differentiation status.

P18, an important regulatory protein of cell differentiation [39, 40], can also affect cell proliferation by adjusting CDK4/6, inhibiting cells from transitioning from the G1 to S phase. To identify the potential relationship of P18 with *CLDN1/7* and *HIF1α*, we next investigated the changes of P18 in NPCs under the hypoxic conditions.

### Hypoxia stimulated changes in P18, which exhibited changes similar to *CLDN7* and HIF1α in NPCs

Hypoxia conditions significantly promoted P18 protein expression in CNE2 cells and inhibited its expression in CNE1 cells, which changed similarly as *CLDN7* and HIF1α in NPCs (Figure 6A). The change was accompanied by a significant reduction of cell proliferation and viability, as demonstrated by EdU and MTT assays.

The analysis of the flow cell cycle was consistent with the change in P18 expression in both cell types. At the initial stage of hypoxia, both cells showed a partial G1 arrest and a decrease in the S phase fraction. With the development of hypoxia, the inhibitory effect on CNE1 was reduced, and the cell proliferation was accelerated (Supplementary Figure S1).

### HIF1α may directly regulate the expression of *CLDN1/CLDN7* and further affect P18 expression through *CLDN7*

Using small interfering RNA transfection technique, we investigated the potential correlation between HIF1α and *CLDN1*, as well as *CLDN7* and P18 in NPCs. The siRNA sequences are shown in Table 2.

**Figure 5:**
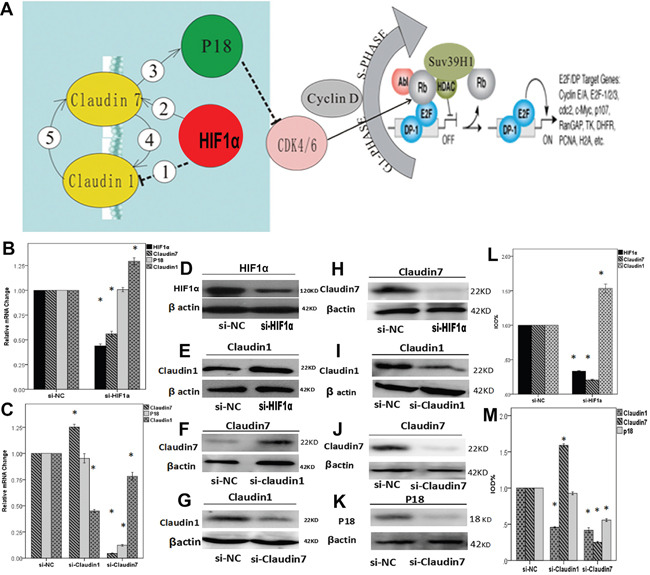
**A**. Diagram showing related genes association in the cell signaling pathway. In NPCs, hypoxia condition enhances the expression of HIF1α and further up-regulate *CLDN7➁*, which consequently stimulates the expression of P18 ➂ and other downstream effectors. HIF1α also down-regulates *CLDN1 ➀* potentially through *CLDN7*. There could be a feedback regulation between *CLDN1* and *CLDN7: CLDN7* positively regulates *CLDN1* ➃, whilst *CLDN1* negatively modulates the expression of *CLDN7* as a negative feedback loop ➄. **B-M**. RT-PCR and western bloting analysis to reveal the interactions between HIF1α, *CLDN1, CLDN7* and P18. (B) Quantification analysis showed that knock down HIF1α could down-regulate the mRNA expression of *CLDN7* but promote *CLDN1* up-regulation. Si-HIF1α can not directly affect the expression of P18. (C) Silencing *CLDN1* enhanced the expression of *CLDN7* and did not affect P18. In comparison, silencing *CLDN7* down-regulated the expression of *CLDN1* and P18. (D) Western blotting result showed that silencing HIF1α triggered down-regulation of HIF1α protein (D&L), up-regulation of claudin1 (E&L), and down-regulation of claudin7 (H&L). Silencing *CLDN1* triggered down-regulation of claudin1 (I&M), and at the same time an up-regulation of claudin7 (F&M). In contrast, silencing *CLDN7* greatly reduced claudin7 protein expression (J&M), and triggered a much reduced claudin1 (G&M) as well as P18 expression (K&M). *S*i-NC is the negative control of silencing the relative genes in CNE2. *: P<0.05, all data were compared to the control.

Knock down of HIF1α in NPCs promoted *CLDN1* expression and inhibited *CLDN7* expression at both the mRNA and protein levels, which may also indirectly affect the expression of P18 through the regulation of *CLDN7*. The silencing of *CLDN7* inhibited the expression of P18, which did not occur after HIF1α or *CLDN1* silencing (Figure 5B & 5C).

### Correlation between *CLDN1* and *CLDN7*

The silencing of *CLDN1* up-regulated the expression of *CLDN7*, whereas the silencing of *CLDN7* inhibited the expression of *CLDN1* (Figure 5C). We used siRNA approach to reveal the causal link between *CLDN1* and *CLDN7*, which demonstrated clearly in a lose-of-function analysis that *CLDN7* knockdown significantly reduced mRNA expression of *CLDN1* (Figure 5C); in contrast, *CLDN1* knockdown triggered *CLDN7* up regulation (Figure 5C). Western blot analysis provided further matching evidence of protein expression pattern between *CLDN1* and *CLDN7*, that *CLDN7* knockdown reduced *CLDN1* protein level (Figure 5G & 5M). In contrast *CLDN1* knockdown triggered upregulation of *CLDN7* (Figure 5F & 5M), potentially through a negative feedback loop.

### Cycling hypoxia can inhibit invasion but promote the proliferation of CNE2 cells

Cycling hypoxia resulting from transient fluctuations in tumor perfusion has received increasing attention in recent years. This was because of the significant influence on treatment resistance displayed by tumor cells as well as the endothelial cells of the tumor vasculature under hypoxia condition [33, 34]. Chronic hypoxia can significantly promote the invasion of CNE2 cells, and this phenomenon may persist in cycling hypoxia conditions.

**Figure 6:**
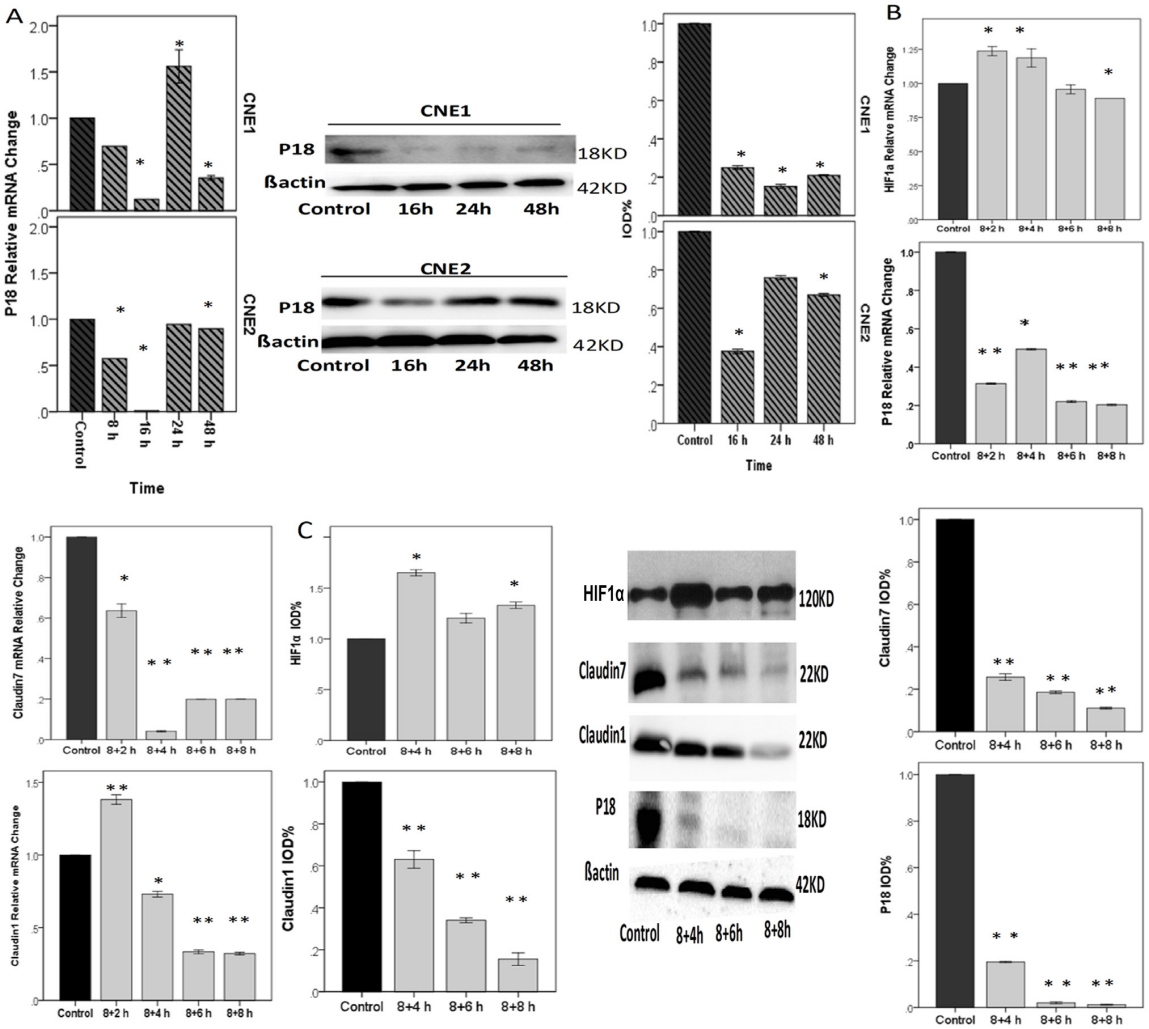
NPCs treated with cycling hypoxia conditions with 8 h hypoxia, followed by culturing in 20%O2 for 2 h, 4 h, 6 h, or 8 h **A**. P18 relative mRNA and protein change in CNE1 and CNE2 cells were decreased. P18 and *CLDN1* had the same trend of expression changes in CNE1 cells (please refere to Figure 4E). In CNE2 cells, p18 declined to the minimum at 16 h and then increased gradually. **B & C**. RT-PCR and Western blotting showed the relative gene and protein expression changes under cycling hypoxia condition. HIF1α relative mRNA change: although we did not find a positive change of reoxygenation to the control, there are differences between 8+2 h and 8+8 h, and between 8+4 h and 8+8 h (p<0.05). P18 relative mRNA change: Compared with the control, cycling hypoxia conditions significantly inhibited the expression of P18. *CLDN1* relative mRNA change was up-regulated at 2 h after reoxygenation, and then significantly declined. *CLDN7* relative m RNA change. Cycling hypoxia conditions inhibited the expression of *CLDN7* dramatically. *: P<0.05; **: P<0.01. All data were compared to the control.

We placed CNE2 cells in a hypoxia condition as shown above, and exposed the cells to cycling hypoxia to further investigate the expression of *CLDNs* and P18 at 4 h, 8 h, and 12 h in CNE2 cells with RT-PCR and western blot analysis. Cycling hypoxia significantly reduced the P18 expression in CNE1 and CNE2 at both mRNA and protein levels (Figure 6A). It also inhibited cell invasion by reducing the expression of *CLDN7* and up-regulating *CLDN1*, and promoted cell proliferation by down-regulating P18 expression (Figure 6B & 6C).

## DISCUSSION

*CLDNs*, as important components of TJs, contribute to the formation of epithelium barriers through interaction with other tight junction proteins. Their function in the development of cancers is mainly due to that some *CLDNs* can also interact with non-tight junction proteins, including the cell adhesion proteins (EpCam and tetraspanins), the signaling proteins (ephrin A and B) and their receptors (EphA and EphB, 32) The expression of *CLDNs* differs in various organs [1, 11, 36], Several articles have reported the correlation between *CLDN1* & *CLDN7* on NPC cells, and demonstrated that CLDN7 may serve as a useful biomarker in the prediction of distant metastasis and patient survival in NPC [24, 25].

To our knowledge, the present study is the first to report the causal link between *CLDNs* and NPC, in close association with the differentiation status of both NPC tissues and cells. Our results showed that poorly differentiated NPC tissue and cells had a high expression of *CLDN7*.

In many epithelial cells, claudin-7 had among the strongest polymerization propensity, claudin-7 is localized not at the tight junction, but on the lateral call membrane, without associated lateral strands [26]. Heiler and colleagues demonstrated that palmitoylated *CLDN7* supports metastasis of HEK cells through association with MMP14 and CD147 [26]. A recently study by Thuma and colleagues further reported that nonpalmitoylated *CLDN7* is required for the formation of intercellular TJs as a TJ protein, as well as inhibiting the tumor metastasis via cytoskeletal keratin and myosin association with EpC, a cancer-initiating cells marker. However, glycolipid-enriched membrane microdomains (GEM)-located palmitoylated *CLDN7* can promote cell motility through association with the cytoskeletal linker proteins actinin, moesin and RhoA, which are engaged in actin cytoskeleton or ganization. *CLDN7* palmitoylation can promote cell invasion by association with uPAR, MMP14 and CD147, and also play a role in epithelial-mesenchymal transition (EMT) of pancreatic adenocarcinoma cells [27]. These studies contribute enormous understanding of the mechanistic role of *CLDN7* in promoting tumor motility and metastasis.

We speculate that *CLDN7* may promote metastatsis of NPC cells based on its palmitoylation status. We showed in this study that knockdown *CLDN7* non-selectively with SiRNA on NPC cells triggered down regulation of both palmitoylated and non-palmitoylated *CLDN7*, as well as a clear reduction of tumor cells metastasis. we speculate that NPC with a high expression of *CLDN7* especially the palmitoylated *CLDN7* may also cause correspondent changes in MMPs to assist the tumor cells invasion, which requires further evidence to approve. We shall further investigate the palmitoylation status and intracellular localisation in the regulation of NPC development and metastasis: once confirmed, palmitoylated *CLDN7* can be used as an indicator of NPC metastasis, and will become a new therapeutic target to inhibit cancer cell invasion and cancer recurrence.

Furthermore, *CLDN1* and *CLDN7* could affect the expression of each other in NPCs: silencing of the *CLDN7* inhibited *CLDN1* expression; in contrast, *CLDN7* expression was significantly up-regulated when *CLDN1* was knocked down, strongly suggesting a potential negative regulatory feedback between *CLDN1* and *CLDN7*. Up-regulated *CLDN1* expression in NPCs confers resistance to cell death [18]. *CLDN1* knockdown promoted the migration related pathway in MKN28 cells, which includes MMP7, TNF-SF10, TGFBR1, and CCL2 [16]. Nm23H1 mediates tumor invasion in esophageal squamous cell carcinoma by regulation of *CLDN1* through the AKT signalling [45]. In consistence with these previous reports, our results suggest that the *CLDN1*-dependent pathway might also be involved in NPC progression.

HIF1α has been commonly recognized as an independent prognostic factor of NPC, since hypoxia could promote NPC progression [37, 38]. By placing NPCs in a 0.1% O_2_ incubator, we mimicked the hypoxic condition similarly as *in vivo* in this study, to reveal the correlation between hypoxia and *CLDN1 / CLDN7*. Hypoxia affects NPC invasion and migration by affecting the expression of *CLDN7*. Our study has shown that knocking down *HIF1α* in NPCs promotes the expression of *CLDN1* and inhibits CLDN7 at protein levels. We speculate that HIF1α may be an upstream activator regulating *CLDN1* and *CLDN7*. If the above speculation is correct, however, both HIF1α and *CLDN1* should decrease at the same time in CNE1 under the hypoxia condition. The opposite phenomenon observed in this study can be explained by the negative feedback regulation between *CLDN1* and *CLDN7*. The reduced expression of HIF1α in CNE1 can promote *CLDN1* and decrease *CLDN7*, whereas the inhibition of *CLDN7* can further down-regulate CLDN1 (Figure 5). Compared with the change of HIF1α, which quickly returned to normal levels after only a transient decrease, the *CLDN7* reduction was more persistently recorded (Figure 4A); therefore, the combined effect is that CLDN1 expression is decreased.

The sensitivity of NPCs to a hypoxic environment is associated with the differentiation status of the cells. Hypoxia can promote the proliferation of CNE1 cells, whilst inhibit CNE2 cell proliferation. P18 is a gene associated with cell differentiation [39, 40] which influences CDK4/6, and inhibits cells transformation from G1 to S phase, thereby affecting cell proliferation. P18 expression in NPCs was consistent with the cell proliferation assay and the flow cytometry analyses (Supplementary Figure S1). However, after silencing *HIF1α*, there was no direct correlation between HIF1α and P18. To our surprise, a decline of *CLDN7* led to decreased expression of P18 (Figure 5). Therefore, we speculate that HIF1α could indirectly affect the expression of P18 by regulating *CLDN7*, thereby affecting cell proliferation and metastasis. In summary, HIF1α may directly regulate the expression of *CLDN1/CLDN7* and further regulate P18 through *CLDN7*.

Cycling hypoxia resulting from transient fluctuations in tumor perfusion has received increasing attention. This is because of the significant influence in treatment resistance displayed by tumor cells, as well as the endothelial cells of the tumor vasculature [33, 34]. In 1996, Kimura et al. demonstrated that in renal cancer tissue intermittent blood cells could pass through the cancer lesions [41]. By testing bleeding in different parts of solid cancers, Hironobu et al. found that the anoxia is mostly due to cycling hypoxia, and the period of anoxia and oxygen richness is determined by the type of cancer tissues and sites [42]. Cycling hypoxia can promote cancer invasion, metastasis and treatment resistance, because once the pressure of hypoxia is lifted, mRNA silencing particles are released from the pressure, leading to excessive translation of certain regulatory proteins [43]. However, in contrary to our expectation, our results showed that cycling hypoxia could inhibit cell invasion by reducing the expression of *CLDN7*. Mathieu et al. proposed that cycling hypoxia could trigger cancer cells reprogramming (iPSC induction) to change their metabolism from oxidative to highly glycolytic status early in the process [44]. We speculate that the change of *CLDN7* may be associated with remodeling and differentiation of NPCs when exposed to cycling hypoxia. Therefore, reoxygenation could potentially increase *CLDN1* expression and decrease P18, suggesting a correlation between these genes.

Our results also suggest that potentially we could use the characteristics of the tumor center and cycling fluctuations of angiogenesis / bleeding to inhibit metastasis and invasion of tumors, by regulating the timing and pattern of cycling hypoxia. If such experiments can be proved *in vivo* in animal models, it may potentially be used as a new treatment for solid tumors, especially those that are not sensitive to radiotherapy.

## MATERIALS AND METHODS

### Cells and reagents

NPC cells CNE1 and CNE2 were purchased from China Tumor Cell Institute, Beijing, China. The following antibodies (Ab) were used: rabbit polyclonal Ab for *CLDN1/CLDN7* (ab15098/ ab27487, Abcam, Cambridge, MA, USA), mouse monoclonal Ab P18 (ab80625, Abcam Cambridge, MA, USA), and mouse monoclonal Ab for HIF1-α (sc-53546, Santa Cruz Biotechnology, INC). Hypoxic conditions were generated using a hypoxia workstation (Ruskin Technologies, UK).

### Immunohistochemistry

Immunohistochemical analysis was performed to evaluate the *CLDN1/CLDN7* protein expression and the correlation between tumor cell differentiation on 66 NPCs samples. The result was analyzed by Pearson’s chi-square test. Written consent was obtained, and ethical approval was granted by the Regional Research Ethics Committee (permission number: 20140993). Primary antibodies against *CLDN1/CLDN7* (1:200) were used. The negative controls were handled in the same way as the experimental samples, except that PBS was applied instead of the primary antibodies. Experiments were performed in accordance with the instructions of the Streptomycin Peroxide Kits (sigma-Aldrich, USA). Brown membranous and/or cytoplasmic staining was classified as positive expression. Immunostaining was observed and imaged under light microscopy. Positive / negative cells and the total cells from minimum five randomly chosen visual fields were counted in each specimen. The quantification was represented by the percentage of positively stained tumor cells, and graded as less than 10% (1+), 10-50% (2+), or more than 50% (3+). The staining intensity was recorded as absent (0), weak (1+), moderate (2+), or strong (3+). The 2 scores were multiplied to generate a final score of 0 to 9 [24].

### Cell culture

The NPC cell lines CNE1 and CNE2 were cultured in 1640 medium supplemented with 10% fetal bovine serum (FBS), 100 U/ml penicillin and 100 μg/ml streptomycin (Life Technologies, Carlsbad, CA, USA) at 37°C and 5% CO_2_ in an incubator. To mimic severe hypoxic conditions, cells were cultured at 37°C in 5% CO_2_, 94.9% N_2_, and 0.1% O_2_ atmosphere in a hypoxia incubator (Ruskin Technologies, UK).

### Cell viability assay

Cell viability was examined with MTT assay according to the manufacturer's instructions. A total of 2×10^3^ cells/well was incubated for24, 48 or 72 h in 96-well plates at a final volume of 100 μl/well. After the addition of 10 μg/well of the MTT reagent, the cells were further incubated for 4-6 h in a 37°C incubator and then agitated for 10 min on a shaker. The cell number was determined based on the standardization from control cells used in the experiment. We then measured the absorbance of the samples at 490 nm (reference wavelength: 690 nm) against the background control, using a 96-well plate reader.

### Cell proliferation assay

Cells were seeded at 2×10^3^ cell/well in poly-D-lysine coated 96-well chamber slides. Two hours prior to the end of the culture, 20-μm EdU was added from a 100 mM 1:10 DMSO/H_2_O stock. Cells were then fixed for 20 min in 4% PFA in PBS, and EdU was detected according to the protocol (KGA 337-100, KeyGEN BioTECH, Nanjing, China). The cell proliferation was quantified as the percentage of EdU incorporating cells against the total number of cells determined by DAPI nuclear staining. Organotypic cultures were sectioned prior to detection of EdU incorporating cells per square millimeter of section, with section area being determined in Image J. The measurement of the detected areas was calibrated across various sizes of organotypic cultures.

### Transwell migration assay

The cell migration assay was conducted using a cell migration assay system (CBA-100, Cell Biolabs, Inc., San Diego, CA, USA) equipped with 8-μm pore size migration chambers [30]. The methods used in this assay were similar to the Matrigel invasion assay, except that the transwell insert was not coated with Matrigel. After 24 h of incubation, the migrated cell numbers were calculated. For each experiment, the number of cells from a minimum of five randomly chosen fields of each filter was quantified, and these experiments were independently performed at least three times.

### Invasion assay

An invasion assay was performed using a BD BioCoat Growth Factor Reduced Matrigel Invasion Chamber (354483, BD Biosciences, San Jose, CA, USA) according to the manufacturer's instructions. Cells were starved in 1640 medium without FBS for 24 h and then plated on the upper insert at 2.5×10^4^/well and incubated in 1640 medium with FBS in 20% or 0.1% oxygen cell chambers, respectively. After a 24-h incubation, the non-invading cells remained on the upper surface of the membrane in each insert were gently removed. Cells that had penetrated through to the bottom surface of the membranes were fixed in 100% methanol and stained with 0.05% Toluidine Blue Solution (206-14555, Wako Pure Chemical Industries Ltd.). For each experiment, the number of cells in seven randomly chosen fields of each filter was quantified, and these experiments were independently performed at minimum of three times.

### Flow cytometry

The cells were incubated in 6-well plates overnight, before synchronized next morning. The cells were treated with nacodazole (Life Technology, USA) in serum free / antibiotics free medium at 150um/ml for 12h [31], then replaced with hypoxia culture condition with fresh culture medium containing 10% FBS and penicillin / streptomycin. Cells were harvested, washed once, and fixed in 75% ethanol at 4°C overnight. The cells were then washed 3 times with PBS, harvested and incubated in PBS suspension containing 5 μg/ml propidium iodide (P3566, Molecular Probes, Eugene, OR, USA) for 30 min at room temperature. The samples were then analyzed with a FACS flow cytometer (BD FACS Calbur TM).

### Quantitative RT-PCR

Total RNA was extracted from the cells using TRIzol (Invitrogen, USA) following the manufacturer’s instructions. One microgram of total RNA was subjected to reverse transcription to synthesize cDNA using the PrimeScript RT™ reagent reverse Kit (TaKaRa, Japan) for 15 min at 37°C and 5 s at 85°C. The PCR program started with an initial denaturation at 95°C for 30 S, followed by 40 cycles (95°C for 5 s, 60°C for 30 s), and 1 μg of cDNA was used for the PCR. HIF1α and Claudin7 were amplified along with β-actin as an internal control following the instructions of the SYBR Premix Ex Taq™ II (TaKaRa, Japan). The PCR reaction conditions and the primer sequences of HIF1α, Claudin-7, and β-actin are shown in Table 1.

### RNA interference by synthetic siRNA

Selective targeting of HIF1α, *CLDN1, CLDN7* and p18 was performed using specific siRNAs. As a control, an siRNA sequence (si-NC) was employed that does not target any gene in the human genome and has been tested by microarray analysis (Dharmacon, Chicago, IL, USA). The siRNAs were synthesized commercially (Biomers.net GmbH, Ulm, Germany). The sequences are shown in Table 2. Transfection of the siRNA (final concentration 100 nM) was performed with Lipofectamine 2000.

### Western blotting

Cells were washed with ice-cold PBS, harvested and lysed in lysis buffer and 0.1% PMSF (KAIJI, Nanjing, China). The protein concentration of the supernatant was determined with a BCA protein assay kit according to the manufacturer's instructions. Proteins were separated by SDS/PAGE and were transferred to polyvinylidenedifluoride membranes (Amersham).

### Statistical analyses

All values are reported as the mean ± SEM from minimum three independent experiments, unless otherwise stated. Two-sided Student's unpaired t test was used for the statistical analyses. ** p<0.01; *, p<0.05.

## SUPPLEMENTARY FIGURE


