# Correction: Cycling hypoxia affects cell invasion and proliferation through direct regulation of claudin1 / claudin7 expression, and indirect regulation of p18 through claudin7

**DOI:** 10.18632/oncotarget.27385

**Published:** 2021-09-14

**Authors:** Hong Liu, Feifei Jiang, Xinshan Jia, Jing Lan, Hao Guo, Erran Li, Aihui Yan, Yan Wang

**Affiliations:** ^1^ Department of Otolaryngology, The First Affiliated Hospital of China Medical University, Shenyang, Liaoning 110001, China; ^2^ Department of Pathology, China Medical University, Shenyang, Liaoning 110001, China; ^3^ Department of Dermatology, China Medical University, Shenyang, Liaoning 110001, China; ^4^ Institute of Respiratory Disease, The First Affiliated Hospital of China Medical University, Shenyang, Liaoning 110001, China

**This article has been corrected:** In the Materials and Methods section, the company who designed and provided the Si-RNA is listed incorrectly. The proper company name is given below. In addition, the value ‘p18’ has been changed to ‘p18’ throughout the paper, including Figures 5 and 6. Lastly, the wrong picture was accidentally selected for the CNE2 Si-Claudin7 invasion (bottom right panel), in Figure 2B. The corrected Figure 2B is shown below. The authors declare that these corrections do not change the results or conclusions of this paper.

Original article: Oncotarget. 2017; 8:10298–10311. 10298-10311. https://doi.org/10.18632/oncotarget.14397

## MATERIALS AND METHODS

### RNA interference by synthetic siRNA

Selective targeting of HIF1α, CLDN1, CLDN7 and p18 was performed using specific siRNAs. As a control, an siRNA sequence (si-NC) was employed that does not target any gene in the human genome and has been tested by microarray analysis (Dharmacon, Chicago, IL, USA). The siRNAs were synthesized commercially (Genephama, Shanghai, China). The sequences are shown in Table 2. Transfection of the siRNA (final concentration 100 nM) was performed with Lipofectamine 2000.

**Figure 2:**
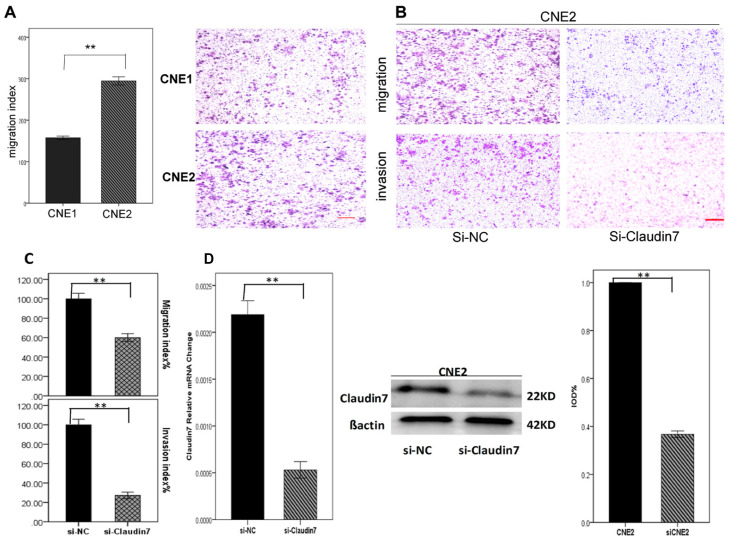
**A.** Migration index analysis of CNE1 and CNE2 using transwell model showed that CNE2 cells with high CLDN7 expression had a significantly increased migration ability than CNE1 cells. **D.** RT-PCR and Western blotting showed the results of knocking down CLDN7 in CNE2; the Si-RNA silencing efficiency was approximately 60-70%. **B, C.** Migration and invasion results of Si-NC and si-Claudin7 in CNE2 demonstrated that knocking down the CLDN7 expression inhibited the migration and invasion ability of CNE2 cells. Si-NC is the negative control of silencing the expression of CLDN7 in CNE2. All statistical analysis were compared to the control. Scale bar = 100μm. **: P<0.01.

**Figure 5:**
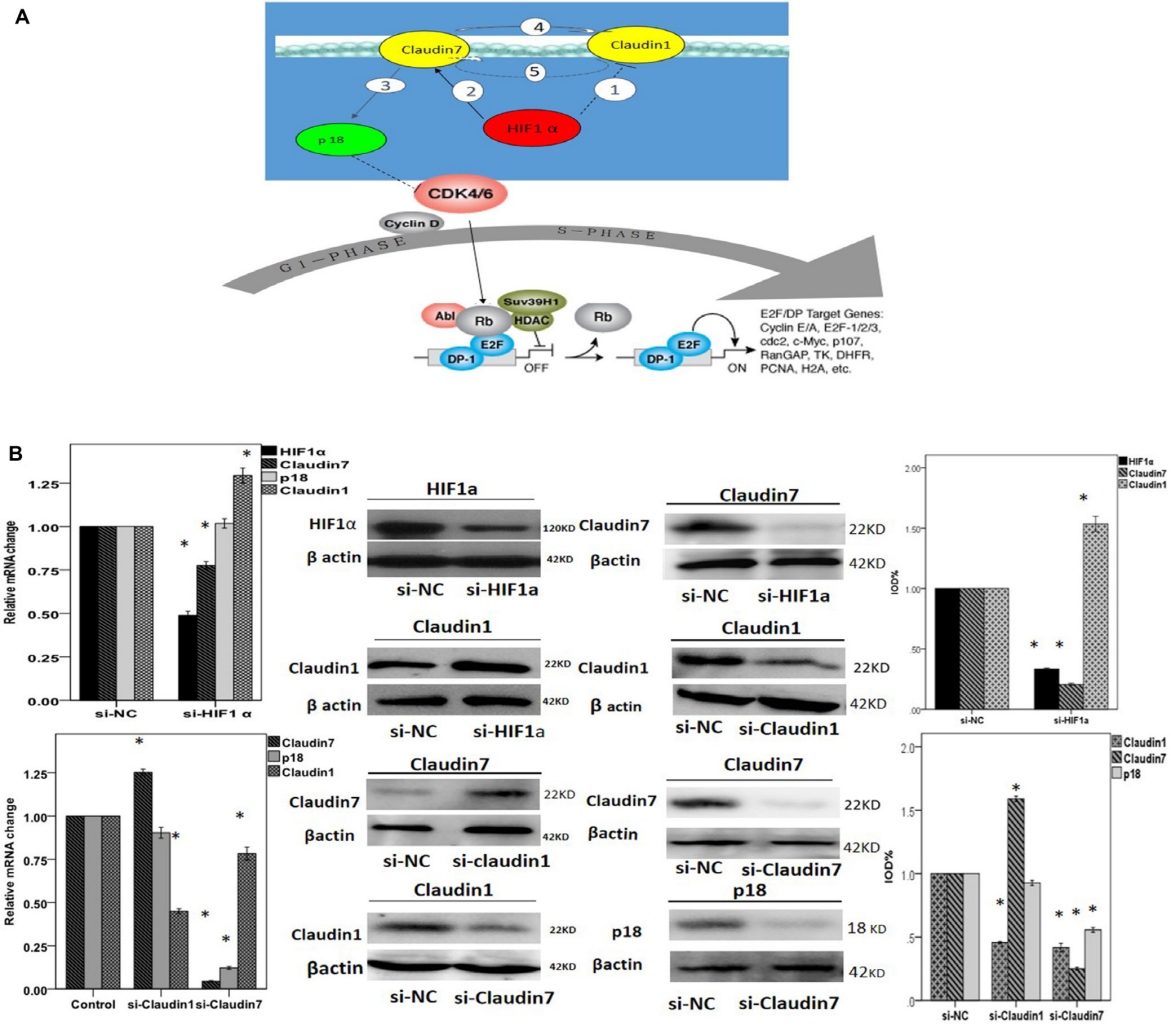
**A.** Diagram showing related genes association in the cell signaling pathway. In NPCs, hypoxia condition enhances the expression of HIF1α and further up-regulate CLDN7②, which consequently stimulates the expression of p18 ③ and other downstream effectors. HIF1α also down-regulates CLDN1 ① potentially through CLDN7. There could be a feedback regulation between CLDN1 and CLDN7: CLDN7 positively regulates CLDN1 ④, whilst CLDN1 negatively modulates the expression of CLDN7 as a negative feedback loop ⑤. **B**-**M**. RT-PCR and western bloting analysis to reveal the interactions between HIF1α, CLDN1, CLDN7 and p18. (B) Quantification analysis showed that knock down HIF1α could down-regulate the mRNA expression of CLDN7 but promote CLDN1 up-regulation. Si-HIF1α can not directly affect the expression of p18. (C) Silencing CLDN1 enhanced the expression of CLDN7 and did not affect p18. In comparison, silencing CLDN7 down-regulated the expression of CLDN1 and p18. (D) Western blotting result showed that silencing HIF1α triggered down-regulation of HIF1α protein (D&L), up-regulation of claudin1 (E&L), and down-regulation of claudin7 (H&L). Silencing CLDN1 triggered down-regulation of claudin1 (I&M), and at the same time an up-regulation of claudin7 (F&M). In contrast, silencing CLDN7 greatly reduced claudin7 protein expression (J&M), and triggered a much reduced claudin1 (G&M) as well as p18 expression (K&M). Si-NC is the negative control of silencing the relative genes in CNE2. *: P<0.05, all data were compared to the control.

**Figure 6:**
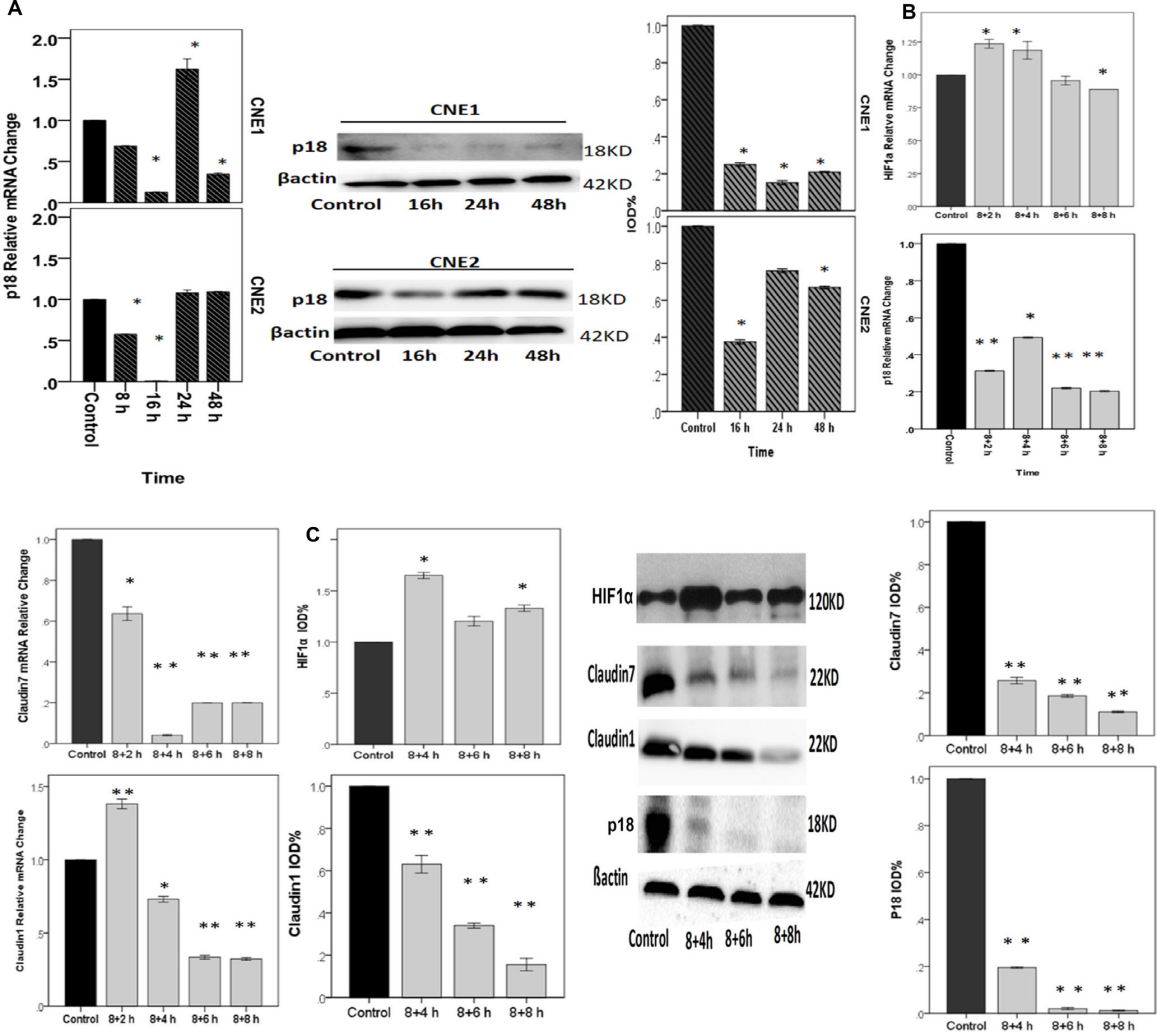
NPCs treated with cycling hypoxia conditions with 8 h hypoxia, followed by culturing in 20%O2 for 2 h, 4 h, 6 h, or 8 h.

**A.** p18 relative mRNA and protein change in CNE1 and CNE2 cells were decreased. p18 and CLDN1 had the same trend of expression changes in CNE1 cells (please refere to Figure 4E). In CNE2 cells, p18 declined to the minimum at 16 h and then increased gradually. **B** & **C.** RT-PCR and Western blotting showed the relative gene and protein expression changes under cycling hypoxia condition. HIF1α relative mRNA change: although we did not find a positive change of reoxygenation to the control, there are differences between 8+2 h and 8+8 h, and between 8+4 h and 8+8 h (p<0.05). p18 relative mRNA change: Compared with the control, cycling hypoxia conditions significantly inhibited the expression of p18. CLDN1 relative mRNA change was up-regulated at 2 h after reoxygenation, and then significantly declined. CLDN7 relative m RNA change. Cycling hypoxia conditions inhibited the expression of CLDN7 dramatically. *: P<0.05; **: P<0.01. All data were compared to the control.

