# Supplementary material for: Cycling hypoxia affects cell invasion and proliferation through direct regulation of claudin1 / claudin7 expression, and indirect regulation of P18 through claudin7
Source: Oncotarget. 2016 Dec 31;8(6):10298–311. doi: 10.18632/oncotarget.14397 (PMC5354660; doi:10.18632/oncotarget.14397)
Supplement: Supplementary file 1 [file oncotarget-08-10298-s001.pdf]

Cycling hypoxia affects cell invasion and proliferation through direct regulation of claudin1 / claudin7 expression, and indirect regulation of P18 through claudin7

SUPPLEMENTARY FIGURE

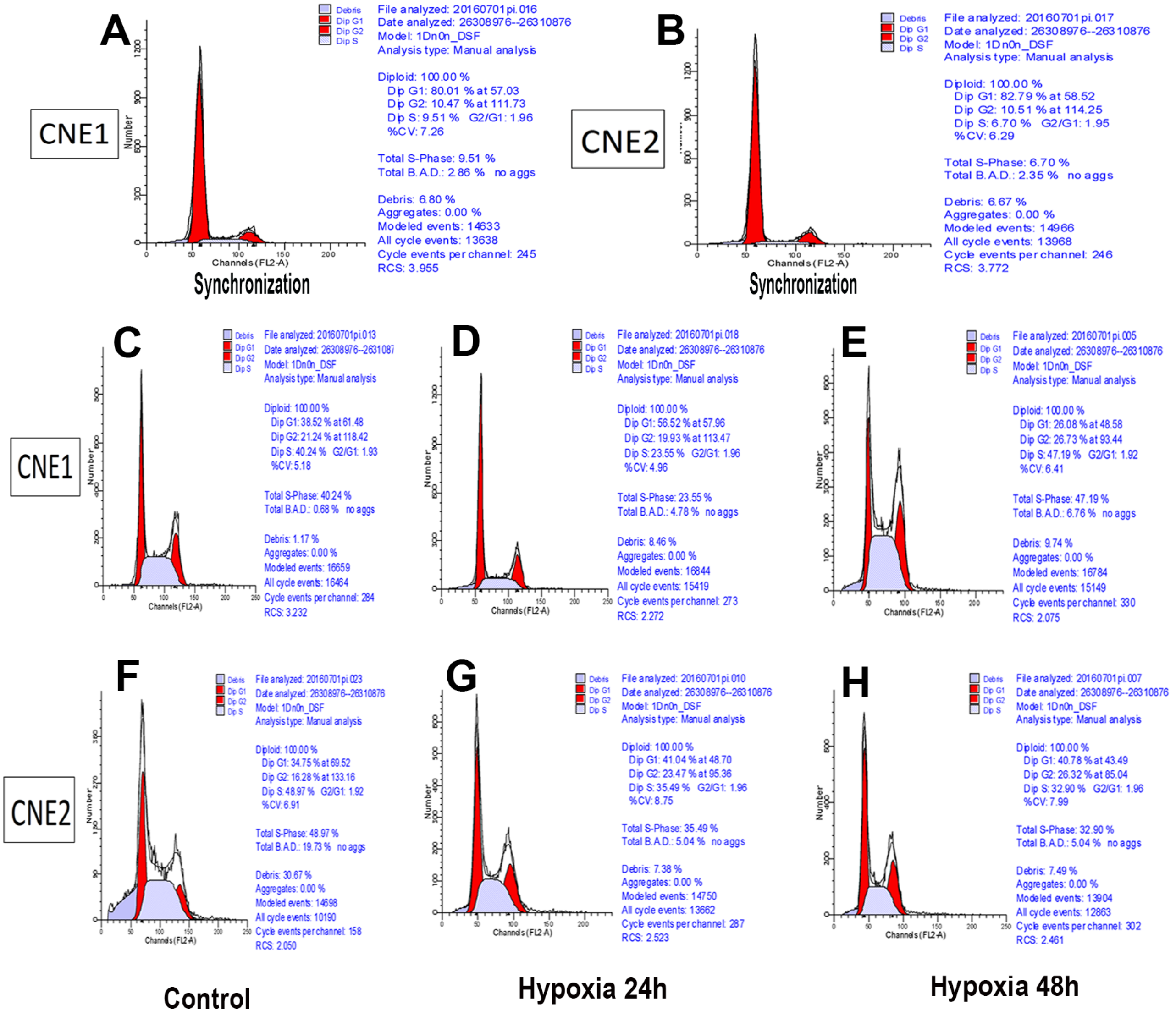

Supplementary Figure 1: The results of the flow cell cycle. A & B. are synchronized CNE1 & CNE2 cells, which showed an obvious arrest at G1 phase compared with the control cells, As shown in **C-E.**, the fraction of cells in S phase was increased in CNE1 cells, and **F-H.** show that CNE2 cells were arrested at G1, which was consistent with the pattern of P18 changes.
